# Kinetoplast-Directed Therapies: A Selective Mitochondrial Approach to Combat Leishmaniasis

**DOI:** 10.3390/ph19040537

**Published:** 2026-03-26

**Authors:** Jenny A. Botero-Buitrago, Juan Camilo Cardozo-Muñoz, David Cisneros, Javier Santamaría-Aguirre, Koraima Torres, Socorro Espuelas, Javier Carrión, Christophe Dardonville

**Affiliations:** 1 Institute of Medicinal Chemistry, IQM-CSIC, Juan de la Cierva 3, 28006 Madrid, Spain; jeboterob@unal.edu.co (J.A.B.-B.); jcamilo.cardozo@iqm.csic.es (J.C.C.-M.); david.cisneros@iqm.csic.es (D.C.); 2 Research Group on Biodiversity, Zoonoses and Public Health (GIBCIZ), Research Institute on Zoonoses (CIZ), Faculty of Chemical Sciences (FCQ), Central University of Ecuador, Quito 170521, Ecuador; jrsantamaria@uce.edu.ec (J.S.-A.); koraimatorres009@gmail.com (K.T.); 3 Department of Pharmaceutical Sciences, School of Pharmacy and Nutrition, University of Navarra, 31009 Pamplona, Spain; sespuelas@unav.es; 4 ICPVet Research Group, Department of Animal Health, Faculty of Veterinary Medicine, Complutense University of Madrid, 28040 Madrid, Spain; 5 Research Institute Hospital 12 de Octubre, 28041 Madrid, Spain

**Keywords:** leishmaniasis, kinetoplast, targeted drug delivery, nanoparticle, nanomedicine, DNA minor groove binder, kDNA, G-quadruplex, mitochondrion-targeted, topoisomerase

## Abstract

The leishmaniases are a group of neglected tropical diseases caused by kinetoplastid protozoa of the genus *Leishmania*, transmitted by phlebotomine sandflies. In the absence of a human vaccine, current chemotherapeutic options remain suboptimal due to limited target selectivity, high cost, restricted availability in endemic low-resource regions, and escalating parasite resistance. This review highlights recent advances in rational drug design directed at the kinetoplast—a distinctive mitochondrial organelle critical for parasite viability. Different targets (e.g., kDNA, G-quadruplex, topoisomerases) and innovative approaches employing mitochondrion-targeted small molecules are discussed, as well as ligand-functionalized nanoparticle delivery systems that can transport bioactive agents to the parasite’s mitochondrial microenvironment. These strategies highlight the kinetoplast’s strong translational relevance as a selective antileishmanial target. By exploiting its unique molecular machinery, these strategies may offer improved parasite selectivity, although potential mitochondrial liabilities in host cells must be carefully evaluated.

## 1. Introduction

The leishmaniases are vector-borne protozoan diseases caused by multiple *Leishmania* species, presenting in three main clinical forms: visceral leishmaniasis (VL), cutaneous leishmaniasis (CL), and mucocutaneous leishmaniasis (MCL). VL is the most severe form, almost invariably fatal if left untreated, whereas CL and MCL cause substantial morbidity, lifelong disfigurement, and social stigma. Globally, an estimated 700,000–1,000,000 new cases occur annually, with CL accounting for the majority of infections; the disease remains endemic in more than 90 countries and disproportionately affects vulnerable populations with limited access to healthcare [1]. Despite this considerable impact, no vaccine is currently available for human leishmaniasis [2]. In contrast, several vaccines have been developed for canine leishmaniasis and are used in endemic regions as part of control strategies. However, vaccine efficacy remains variable and current research continues to focus primarily on improving chemotherapeutic options.

Kinetoplastids are a group of flagellated protozoa distinguished by the presence of the kinetoplast, a unique mitochondrial organelle that contains an intricate, catenated network of mitochondrial DNA (kDNA). Within the order Kinetoplastida, the family Trypanosomatidae is of public health relevance, as it includes *Leishmania* spp., *Trypanosoma cruzi*—the causative agent of Chagas disease—and *Trypanosoma brucei*, responsible for African trypanosomiasis, all of which are major pathogens associated with neglected tropical diseases [3]. *Leishmania* parasites alternate between two major developmental stages during their life cycle: the flagellated promastigote form that develops in the sand fly vector and the intracellular amastigote form that replicates within mammalian macrophages. These stages differ markedly in morphology, metabolism, and susceptibility to therapeutic agents. In addition, clinically relevant species such as *Leishmania donovani*, *Leishmania infantum*, *Leishmania major*, *Leishmania tropica*, and *Leishmania braziliensis* show important differences in geographic distribution, pathogenicity, and drug susceptibility profiles [4]. Such biological diversity has important implications for therapeutic development, particularly for strategies targeting parasite-specific organelles such as the kinetoplast and mitochondrion [5]. Current therapies against leishmaniasis and other kinetoplastid diseases remain suboptimal, challenged by increasing resistance, dose-limiting toxicity, prohibitive cost, and inconsistent availability. Therefore, deciphering parasite biology and elucidating drug–parasite interactions at both molecular and cellular levels are essential to identify novel targets and drive the development of next-generation antileishmanial agents [6].

## 2. Drawbacks of Current Chemotherapy of Leishmaniasis

**Figure 1:**
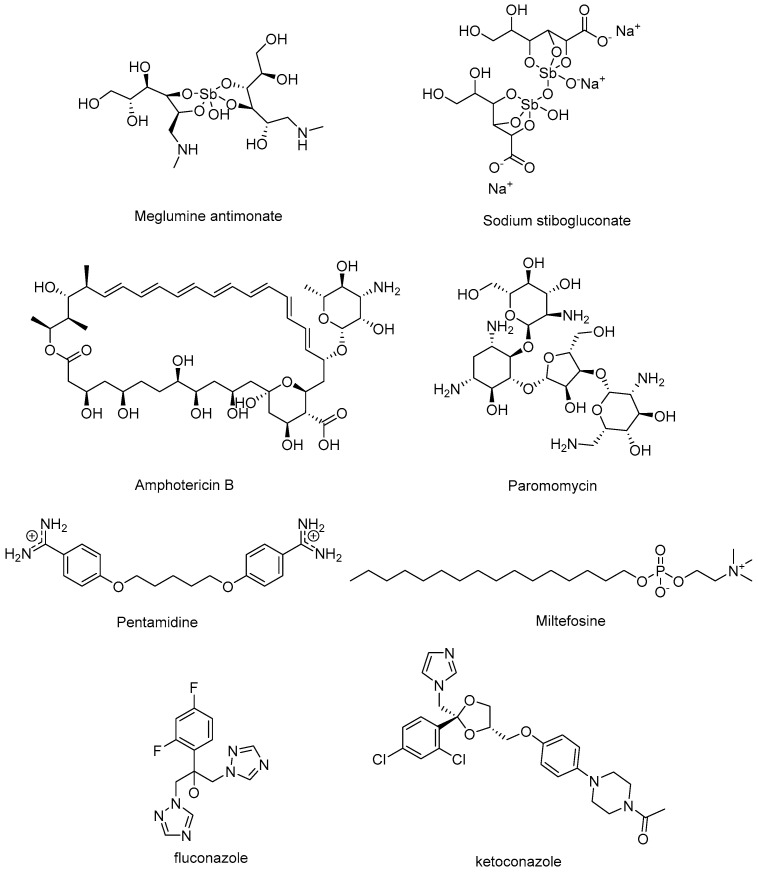
Chemical structures of drugs used for the treatment of leishmaniasis. These include compounds approved by regulatory agencies such as the U.S. Food and Drug Administration (FDA), the European Medicines Agency (EMA), and national authorities in endemic countries, as well as drugs recommended by the World Health Organization (WHO) for clinical use.

Chemotherapy remains the cornerstone of leishmaniasis management, although the position of each drug in therapeutic algorithms varies depending on the clinical form of the disease. Pentavalent antimonials (Figure 1) have historically been the standard of care and continue to represent the first-line option for CL and MCL in many endemic regions. In contrast, in VL, their use has shifted to a second-line role in areas such as the Indian subcontinent, where high levels of resistance have rendered them largely ineffective [7]. Conversely, the liposomal formulation of amphotericin B (AmBisome^®^, Gilead Sciences Inc., Foster City, CA, USA) is considered the first-line therapy for VL owing to its superior efficacy and reduced toxicity; however, in CL and MCL it is generally reserved as a second-line option for refractory or severe cases, since its high cost, limited availability, and requirement for cold-chain storage restrict widespread use in many endemic regions. Additional agents include oral miltefosine (MF) [8], effective against VL and CL but contraindicated in pregnancy due to teratogenicity, as well as paromomycin and pentamidine, which serve as alternatives in specific settings. The antifungal drugs fluconazole and ketoconazole (Figure 1) are other treatment options for CL [9]. Nevertheless, across all forms of the disease, current regimens remain constrained by significant obstacles [10,11]. Drug toxicity leading to systemic adverse events such as cardiotoxicity, hepatotoxicity, nephrotoxicity, pancreatic dysfunction, and gastrointestinal disorders is the most prominent one (Table 1) [12,13,14,15]. Variable efficacy across endemic regions, complex administration schedules, and the growing problem of resistance is another issue of leishmaniasis chemotherapy [16,17,18]. Other limitations include a low or variable bioavailability, poor solubility, limited and poorly characterized tissue distribution, high protein binding, or short half-lives for paromomycin and pentavalent antimonials. Such characteristics result in rapid drug clearance and subtherapeutic plasma levels, ultimately necessitating prolonged treatment regimens that compromise patient adherence and increase the risk of cumulative toxicity [19].

Additional chemical scaffolds have been explored and a few clinical leads [20], which were identified through phenotypic screens [i.e., the proteasome inhibitors GSK34942459 and LXE-408, the CDC-2-related kinase 12 (CRK12) inhibitor DNDI-6899 (GSK3186899), the oxaboroles DNDI-6174 and DNDI-6148, and the nitroimidazole DNDI-0690], have progressed to early stages of clinical development (reviewed by Ebiloma et al.) [16,21]. However, the last drugs approved for human VL, miltefosine and paromomycin, were introduced in 2002 and 2006, respectively [22].

Collectively, these shortcomings underscore the urgent need to identify vulnerable parasitic targets—molecules essential for parasite survival or virulence, ideally expressed in clinically relevant stages—to develop safer, more affordable, and more effective antileishmanial therapies [3].

**Table 1:** Summary of key pharmacological parameters—including route of administration, dosing regimen, therapeutic efficacy, and principal limitations—for conventional antileishmanial drugs. References provided correspond to clinical and experimental data sources.

Drug	Administration	Regimen	Efficacy	Limitations	References
Pentavalent antimonials: - Meglumine antimonate - Sodium stibogluconate	intravenous (IV) or intramuscular (IM)	20 mg/kg/day for 30 days	35–95% (Region- and Strain-Dependent)	Painful injections Cardiotoxicity Nephrotoxicity Pancreatitis	[23]
Liposomal Amphotericin B	IV	3–5 mg/kg over 6–10 infusions	60–85% in Africa >95% in India	Rigor and chills Nephrotoxicity Hypokalemia Anaphylaxis	[24,25,26,27,28]
Paromomycin	IM	11 mg/kg/day for 21 days	63–85% in Africa 94–95% in India	Painful injections Nephrotoxicity Ototoxicity	[29,30,31]
Miltefosine	oral	2.5 mg/kg/day for 28 days	≈ 85% in Africa 94% in India	Teratogenicity Nephrotoxicity Gastrointestinal toxicity	[32,33,34]
Pentamidine	IM or IV	4 mg/kg/day for 7–10 days	Variable Mostly for CL	Painful injections Nephrotoxicity Hypoglycemia Cardiovascular toxicity	[35,36,37]

## 3. Key Structural Components of Leishmania: Kinetoplast Biology and Drug-Target Potential

Trypanosomatids of greatest concern to the WHO—including *Leishmania* spp., *T. cruzi*, and *T. brucei*—possess a distinctive mitochondrial organelle known as the kinetoplast, which harbors their mitochondrial DNA (kDNA). This specialized structure presents an attractive target for therapeutic intervention due to its unique composition and critical biological functions [38]. Identifying suitable therapeutic targets against *Leishmania* requires first an understanding of their life cycle, anatomical structures, and biological functions. These parasites alternate between two main morphological stages: the promastigote, an elongated and motile form (~5–20 µm) that develops in the sand fly vector and is characterized by a long anterior flagellum, and the amastigote, a smaller (~6–8 µm), ovoid, intracellular form with a rudimentary flagellum, specifically adapted to survive and replicate within mammalian macrophages [39]. Both forms are surrounded by a glycocalyx composed of stage-specific glycoconjugates, among which lipophosphoglycan (LPG) is the most prominent. In promastigotes, LPG facilitates attachment to the sand fly midgut, shields the parasite from complement-mediated lysis, and modulates host phagocytosis [40,41]. LPG expression is significantly downregulated in amastigotes, which instead rely on other surface molecules for immune evasion and intracellular survival [42,43]. A distinctive and evolutionarily conserved organelle in both promastigote and amastigote forms is the flagellar pocket, a plasma membrane invagination at the base of the flagellum that serves as the exclusive gateway for endocytosis and exocytosis. This specialized compartment is enriched in LPG and glycoprotein GP63, orchestrating vesicular trafficking, modulating host immune responses, and directing parasite orientation toward phagocytic cells to promote uptake [44]. As mentioned before, a defining feature of *Leishmania* is the kinetoplast, a unique mitochondrial organelle located adjacent to the flagellar basal body. It contains the parasite’s mitochondrial genome, arranged as a highly compact network of maxicircles (~20–40 kb) and thousands of heterogeneous minicircles (0.5–10 kb) concatenated into a disk-shaped structure [45]. Maxicircles encode mitochondrial rRNAs and essential subunits of the respiratory complexes, whereas minicircles provide the guide RNAs required for the extensive RNA editing that characterizes trypanosomatid gene expression. The kinetoplast DNA (kDNA) can constitute a substantial fraction of the total cellular DNA, underscoring its structural and functional significance in parasite survival and adaptation [46].

Overall, the unique structural features and essential functions of the kinetoplast make it a highly attractive and promising pharmacological target for the treatment of leishmaniasis. Scientific studies have highlighted the potential of targeting the kinetoplast due to its crucial role in parasite survival and its distinctive architecture. Disrupting kinetoplast integrity or its replication mechanisms can cause parasite death by impairing mitochondrial functions. The enzymes involved in kinetoplast replication, especially topoisomerases, are sufficiently different from their host counterparts, allowing for selective inhibition and a reduced risk of host toxicity. This strategy offers several advantages: the high specificity of kinetoplast enzymes minimizes off-target effects, and interfering with such a vital organelle is likely to produce a significant antiparasitic impact [38]. Therefore, drugs aimed at kinetoplast components, such as topoisomerases [47], may lead to highly effective and selective antileishmanial therapies, opening new options for the development of effective treatments. Another validated and selective drug target functionally linked to the kinetoplast, though not physically localized within it, is trypanothione reductase (TryR). By maintaining the parasite’s thiol–redox balance, TryR plays a pivotal role in safeguarding kinetoplast integrity against oxidative stress [48]. Novel chemotypes have recently been identified that inhibit *Leishmania* TryR with high selectivity, leading to marked reductions in intracellular amastigote viability [49,50,51].

Post-genomic analyses reveal a reduced but life-stage-adapted mitochondrial genome in *Leishmania* and *Trypanosoma*. Key processes—electron transport, membrane potential (MMP) maintenance, calcium homeostasis, and apoptosis induction—have been probed using purified parasite mitochondria. The relevance of *Leishmania* mitochondrion as drug target is evidenced by the number of clinically used drugs (e.g., amphotericin B, pentamidine, artemisinin, atovaquone) and investigational antileishmanial compounds (e.g., MitoTam [52]) that exert their effect by collapsing MMP and inhibiting respiration. Benzophenone bisphosphonium salts are another example of compounds that selectively target complex II of the electron transport chain in *Leishmania*, causing mitochondrial swelling, ATP depletion, and MMP loss [53]. Among natural products, berberine induces ROS-mediated apoptosis by inhibiting complexes I–III and depleting ATP, whereas flavonoids (luteolin, quercetin) promote kDNA loss and topoisomerase II inhibition [54,55]. Topoisomerase inhibitors further compromise kinetoplast replication and mitochondrial integrity. These multifaceted approaches underscore the translational relevance of targeting mitochondrial and kinetoplast biology and support the development of multitargeted therapies to enhance efficacy and control parasite resistance [56]. However, despite its potential, the kinetoplast poses significant challenges as a therapeutic target, mainly due to its intracellular localization within the parasite’s single mitochondrion.

## 4. Therapeutic Strategies Directed at the Kinetoplast

Adopting a target-based approach to identify new antileishmanial hits is a promising strategy as long as the target is validated based upon essentiality (e.g., gene, DNA, RNA,…) in addition to other parameters such as potential druggability, the availability of structural information, and, preferably, the absence of a similar target in the host to avoid possible risks of host toxicity [57]. Different targets of interest have been studied in recent years (reviewed by Marín et al. [21] and Singh et al. [58]). These include the proteasome, which in kinetoplastids can be distinguished from other eukaryotes, protein kinases, the endonuclease cleavage and polyadenylation specificity factor 3 (CPSF3), the purine salvage pathway, and the mitochondrial DNA (kDNA) [59,60,61]. In trypanosomatid parasites, kDNA replication is a critical process that occurs prior to the nuclear S phase [62]. Since the mitochondrial genome maintenance is essential for parasite survival [62], compounds that target mitochondrial DNA and interfere with kDNA replication are potentially active against kinetoplastid parasites.

### 4.1. DNA Minor Groove Binders

The kinetoplast is made up of thousands of DNA circles topologically linked in a disk-shaped, planar array. It contains two types of circular DNA, the maxicircles that code for rRNA and mitochondrial proteins, and thousands of small DNA minicircles that code for guide RNAs, which are used to edit the mRNA from maxicircles. The extensive AT sequences present in kDNA minicircles produce curved double-helical structures [63,64] that are potential targets for AT-specific DNA minor groove binders (MGBs) [65,66]. Given that kinetoplastid mitochondrial DNA exhibits a high A-T content compared to other organisms, it has been validated as a good target for MGBs.

#### 4.1.1. “Classical” (Di)cationic Minor Groove Binders

**Figure 2:**
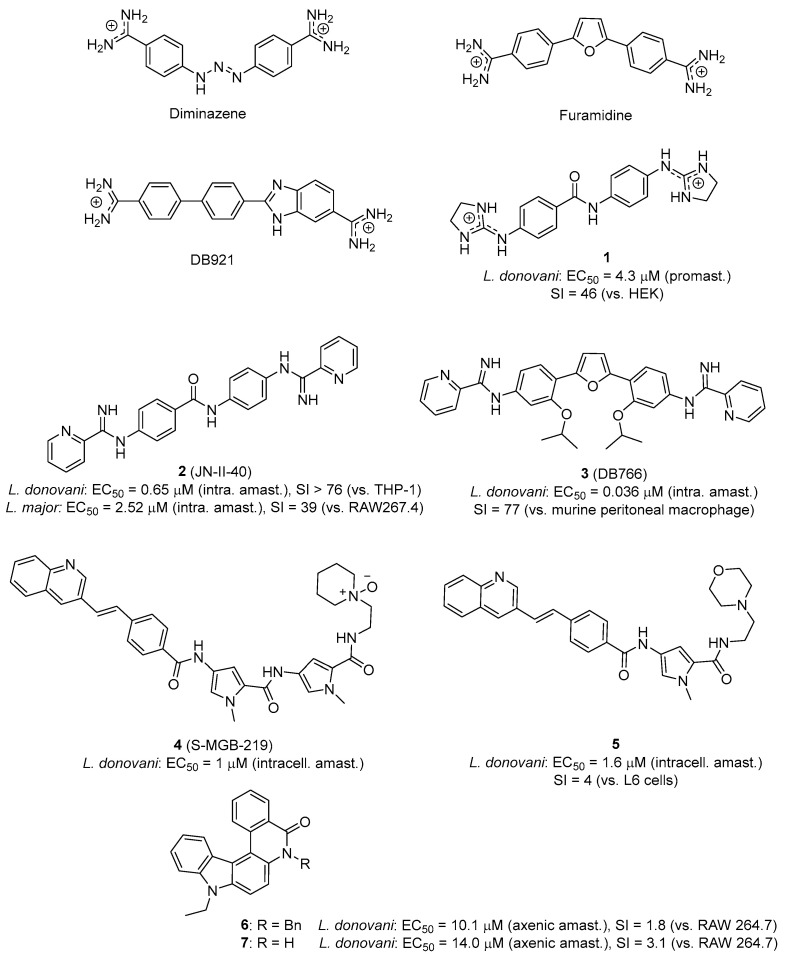
Examples of DNA minor groove binders active against kinetoplastid parasites.

More than three decades ago, Shapiro and Englund showed in *T. brucei* that the diamidines pentamidine and diminazene (Figure 1 and Figure 2) generate minicircle–protein cleavable complexes specific to kDNA [67]. More recently, diminazene was also shown to impair kDNA topology and replication in *T. cruzi* epimastigotes, reducing parasite proliferation without affecting its viability [68].

Aromatic diamidines (e.g., diminazene, furamidine, Figure 2) are dicationic MGBs that bind preferentially to the minor groove of kDNA at AT sites of four or more base pairs and have a well-established antiparasitic activity [65,69,70,71]. In general, the concave shape of MGBs closely matches the convex minor groove shape. However, this crescent shape is not a strict requisite of MGBs because, in some cases, water can mediate the interaction between the minor groove and linear dicationic compounds via hydrogen bonds, hence forming a flexible linker resulting in an isohelical complex [71,72]. This is the case, for instance, in the dicationic near-linear biphenyl benzimidazole DB921 (Figure 2) [73,74]. Over the last two decades, new analogs of furamidine showing a strong binding to the DNA minor groove have been developed. In these analogs, the phenyl groups and the furan ring linker were replaced by heterocycles including thiophene, selenophene, indole or benzimidazole, enhancing the antiparasitic activity, both in vitro and in vivo, compared to pentamidine and furamidine [71,75].

Other classes of dicationic minor groove binding compounds such as bisguanidines and bis(2-aminoimidazolines) (**1**) [76,77] are also very effective against *T. brucei* [78,79,80,81,82,83,84], whereas bisarylimidamides (e.g., **2**, **3**; Figure 2) are more potent against the intracellular parasites *T. cruzi* [85,86,87,88,89] and *Leishmania* [90,91,92,93,94,95,96]. Converting amidines to arylimidamides (AIAs), which also bind to AT-DNA sequences in a structure-dependent manner, is an effective strategy to boost the activity against *Leishmania.* In these compounds, the DNA binding strength varies with substituent size, charge and polarity [97].

The selectivity of MGBs towards kinetoplastid parasites is driven by two main factors: the presence of cationic charges in the molecule favors their accumulation in the mitochondrion of the parasite (driven by the strong inside-negative electrostatic membrane potential) and also promotes preferential binding to AT sites of kDNA. The increased negative electrostatic potential at the floor of narrow AT-containing minor grooves is one of the factors that drives this preferential binding. As a result, these compounds accumulate in the mitochondrion of kinetoplastid parasites (although most mechanistic evidence has been obtained in *Trypanosoma* models), possibly disrupting replication and transcription processes by the inhibition of DNA-dependent enzymes (e.g., topoisomerases, polymerases, nucleases, and helicases) and/or compound-induced conformational changes that disrupt the kinetoplast structure [98,99].

Millan et al. have shown in *T. brucei* that the *N*-phenylbenzamide-based bis(2-aminoimidazoline) compound **1** (Figure 2), which is 100% curative in mouse models of African trypanosomiasis by oral dosage [81,100], alters the integrity of the kinetoplast and disrupts the replication of *T. brucei* kDNA [101]. Interference with the mitochondrial ‘high mobility group’ (HMG) box-containing protein TbKAP6 [102], which is essential for kDNA function, was suggested as possible mode of action [101]. In contrast, most bis(2-aminoimidazolines) are inactive or weakly active against *Leishmania* and *T. cruzi* intracellular amastigotes. This can be attributed to cell uptake differences because a drug targeting kDNA must cross four biological membranes to reach its target. Although the high polarity and basicity (i.e., all have p*K*_a_ values > 9) of bis(2-aminoimidazolines) and related dicationic drugs (e.g., diamidines, bisguanidines) limit their diffusion across biological membranes, transporter-mediated uptake and/or binding to kDNA can influence selective cellular accumulation (see above) [103]. In fact, diamidines are known to act selectively on trypanosomes because of exclusive transport mechanisms that are absent in *Leishmania* species [103,104].

Bisarylimidamides (bisAIA) display distinct physicochemical properties with lower p*K*_a_ (i.e., in the range 4.2−8.4) and higher logP values than diamidines, bisguanidines and bis(2-aminoimidazolines) [90]. They are usually more active against intracellular parasites, although their mode of action against *T. cruzi* and *Leishmania* is still a matter of controversy. This class of compounds binds to the DNA minor groove with more or less specificity depending on the scaffold. Daliry et al. showed that, in *T. cruzi*, the biological activity of very potent bisAIA compounds such as DB766 (**3**, Figure 2) produced deep alterations of the parasite kDNA topology. However, the study showed that the trypanocidal activity of this class of compounds did not correlate with the binding affinity to *T. cruzi* kDNA [105]. A lack of correlation between DNA binding affinity and antikinetoplastid activity was also observed with the structurally related compounds **1** and **2** [106]. Hence, it appears that other targets apart from kDNA may be considered to explain the antiparasitic action of bisarylimidamides. The cytochrome P450 (CYP5122A1) is an example of a target associated with ergosterol metabolism that could be implicated in the antileishmanial action of bisAIA [93].

In addition to the recognition of AT sequences, and to expand the therapeutic potential of DNA MGBs, the recent literature highlights the possibility of engineering diamidines for the sequence-specific recognition of mixed AT/GC base pairs at the DNA minor groove [72,107]. Thus, chemical approaches adding new mixed bps DNA binding motifs could be used to target the compounds to a specific kDNA sequence.

Strathclyde minor groove binders (S-MGBs) are another class of compounds showing antiparasitic activity against *Leishmania donovani* and against various infective agents such as bacteria, viruses, or fungi [108]. These compounds are based upon the structure of the natural product distamycin [109], in which the cationic amidine tail can be replaced by a neutral group—including an aromatic ring—and one of the amide links, can be replaced with the isosteric alkene linker. Perieteanu et al. have reported that an *N*-oxide derivatization of the tertiary amine tail can enhance the selective anti-leishmanial activity of S-MGBs, as exemplified by compound **4** (Figure 2) [110]. The reduction in the overall length of the molecules was also proposed to improve selectivity towards parasitic organisms, such as *Trypanosoma* spp. and *Leishmania* spp, and reduce the likelihood of the molecular aggregation of this class of compounds (e.g., **5**; Figure 2) [111].

#### 4.1.2. Non-Cationic Minor Groove Binders

Phenanthridines are nitrogenous heterocyclic compounds that can be found in many natural products and exhibit different pharmacological properties [112]. It is known that the binding of these compounds with DNA, either via classical intercalation or minor groove binding, depends on the substituents decorating the phenanthridine core [113,114]. Recently, a class of non-cationic DNA MGBs with a “hybrid” structure related to phenanthridine (i.e., indolo [3,2-a]phenanthridine derivatives **6** and **7**, Figure 2) were found active against *L. donovani* in the micromolar range, though with limited selectivity (SI < 5). The compounds were shown to modulate the cell cycle in *Leishmania* parasites [115].

#### 4.1.3. AT-Hook Proteins as Target

The “AT-hook” is a DNA-binding domain of DNA-regulating proteins that interacts in the minor groove of AT-rich sequences. It has been described in the High-Mobility Group A (HMGA) protein family and in different transcription factors and chromatin proteins [106,116,117]. Upon binding, the AT-hook causes bends in the DNA structure through the electrostatic and hydrophobic contacts of the neighboring basic residues of the central RGR core, which fits into the narrow minor groove of the DNA [118]. Since HMGA proteins are involved in a variety of cellular processes in eukaryotes, including kinetoplastid parasites, inhibitors of AT-hook binding proteins such as small-molecule MGBs could provide a therapeutic potential as antiparasitic agents [101,106].

The crystal structure of the HMGA AT-hook 1 domain bound to the minor groove of a DNA oligonucleotide has been reported recently [106]. Small-molecule DNA MGBs with antikinetoplastid activity such as **1** and **2** (Figure 2) were able to inhibit the interaction between AT-hook proteins and AT-rich DNA [101,106]. In particular, **2** is a bisarylimidamide lead compound that shows very promising activity in vitro [90,119] and in vivo ([120], Nué-Martinez et al., unpublished) against *L. donovani*, *Leishmania infantum*, and *Leishmania major*. Based on these results, we can envision that AT-hook proteins such as LamAT-Y, expressed in *Leishmania* parasites [121], are potential targets for this class of compounds [106].

#### 4.1.4. Topoisomerase Inhibitors

Several proteins are involved in kDNA replication (reviewed by Amodeo et al. [62]), many of which are potential targets for antiparasitic chemotherapy. DNA topoisomerases of kinetoplastid parasites have been proposed as antiparasitic targets because they are structurally different from human topoisomerases, thus enabling selectivity towards the parasite [47,122,123]. These enzymes are crucial to maintain the dynamic structure of cellular DNA. They perform essential functions associated with the topological organization of kDNA, participating in the decatenation of minicircles prior to their replication for their subsequent recatenation in the kinetoplast network and contributing to the structural stability of the network during replicative processes [124]. In contrast, the function of host topoisomerases is limited to the topological modulation of non-catenated DNA and they have structural differences from those associated with the Kinetopolastida order. These functional and structural differences, combined with the specific intracellular location of the kinetoplast and the parasite’s absolute dependence on a single mitochondrion, justify the potential of these enzymes as selective therapeutic targets.

DNA topoisomerases (Topo) are divided into two main categories: they transiently cleave one strand of DNA (TopoI) or both strands (TopoII), and they transfer one DNA strand through the other from the same (TopoI) or different DNA molecules (TopoII) [47,122]. In trypanosomatids, the mitochondrial TopoII, encoded by kDNA, is essential, and a failure of this enzyme leads to the disruption in the replication of minicircles, loss of kDNA network, and death of the parasites [122,124,125]. Different classes of molecules have been reported as DNA topoisomerase inhibitors. Class I inhibitors (“topoisomerase poisons”) stabilize the DNA-topoisomerase covalent complex known as TOPcc, which causes genomic instability, leading to cell cytotoxicity. On the other hand, class II inhibitors (“catalytic inhibitors”) block the enzyme’s active site preventing enzyme–DNA interactions and interfering with the enzyme catalytic function [47].

**Figure 3:**
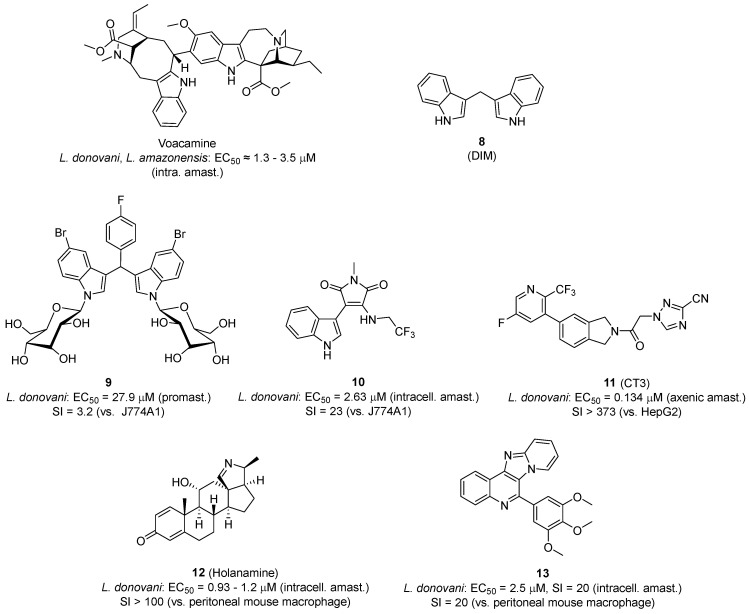
Examples of topoisomerase inhibitors active against *Leishmania* and other kinetoplastid parasites.

Several TopoIB inhibitors active against *Leishmania* have been reported in the literature (reviewed by Saha et al. [123]). For example, voacamine, an indole alkaloid isolated from the plant *Tabernaemontana coronaria*, is a parasite-selective uncompetitive *Ld*TopoIB-specific poison that is effective in vitro against wild-type and drug-resistant isolates of *L. donovani*, *Leishmania amazonensis*, and *T. cruzi* (Figure 3). In a BALB/c mice model of VL, voacamine cleared the parasite burden in spleen and liver at an intraperitoneal dosage of 5 mg/kg [126].

Diindolylmethanes have also shown promising results against *Leishmania* parasites. (3,3′)-diindolylmethane (DIM) (**8**, Figure 3), a putative DNA MGB [127], is a natural compound that inhibits tumor growth in human cell lines and in vivo models. DIM is a non-competitive class I inhibitor of TopoI of *Leishmania donovani* (IC_50_ = 1.2 μM). It was suggested that the stabilization of the topoisomerase I–DNA cleavable complex formation hinders the DNA relaxation activity and causes the inhibition of parasite replication and transcription [128,129].

Kour et al. have shown that the glycosylation of 3,3-DIM analogues active against *L. donovani* promastigotes, but devoid of TopoIB inhibitory activity, resulted in reduced cytotoxicity against macrophages without affecting the anti-leishmanial activity. These glycosylated analogs (**9**, Figure 3) inhibited recombinant *Ld*TopILS in the high micromolar range, suggesting that glycosylation is an interesting approach to target TopoI inhibitors to *Leishmania* parasites [130].

Indolylmaleimide derivatives are another class of antileishmanial compounds designed as *Ld*TopILS inhibitors. Das et al. showed that compound **10**, which is active against *L. donovani* intracellular amastigotes in the low micromolar range (IC_50_ = 2.63 µM), inhibits *Ld*TopILS at 800 µM concentration [131]. The results of this study suggest that this compound is a class II inhibitor; however, the two orders of magnitude difference between the cellular and the enzymatic activities indicates that other targets (in addition to *Ld*TopILS) may be involved in the antileishmanial activity of **10**.

Compound CT3 (**11**, Figure 3) is an example of irreversible inhibitor of trypanosomal TopoII that stabilizes the covalent DNA–enzyme complex, which leads to dsDNA breaks. CT3 proved to be a very potent trypanocidal compound both in vitro and in mouse models of Chagas disease and human African trypanosomiasis. The compound was also active against axenic amastigotes of *L. donovani* (IC_50_ = 0.134 µM) [132].

Holanamine (**12**, Figure 3), a plant-derived steroidal alkaloid, has shown potent in vitro antileishmanial activity with IC_50_ = 2.66 and 3.80 µM against WT and multi-drug-resistant *L. donovani* promastigotes, respectively [133]. The compound inhibits the catalytic activity of *L. donovani* topoisomerase 1B (*Ld*TopIB) in a noncompetitive manner (IC_50_ = 2.81 μM), without inhibiting the catalytic activity of human TopoI.

The pyrido [2,1′:2,3]imidazo [4,5-c]quinoline derivative **13** is a class I inhibitor that stabilizes the *Ld*Top1−DNA covalent complex and inhibits TopoI religation activity, leading to apoptosis-like cell death in drug sensitive and antimony-resistant *L. donovani* clinical isolates. This compound showed in vivo antileishmanial activity in a mouse model of visceral leishmaniasis [134].

Other natural product derivatives such as terpenyl-quinones, lignans (niranthin) [135], lignan glycosides (lyoniside and saracoside) [136] and naphthyridines [137] have also shown potent anti-leishmanial activity by preventing the relaxation of supercoiled DNA, impairing the enzyme function. All these examples illustrate nicely the potential of parasite topoisomerases as a target for antikinetoplastid drugs. However, as mentioned by Kour et al. [47], many *Leishmania* inhibitors have been described to date, but there are not any drugs targeting leishmanial topoisomerases yet in clinical trials. Therefore, it is crucial to further investigate these promising compounds and their mechanisms of action in order support the rational development of chemotherapeutic strategies against leishmaniasis.

### 4.2. G-Quadruplex Stabilizers

**Figure 4:**
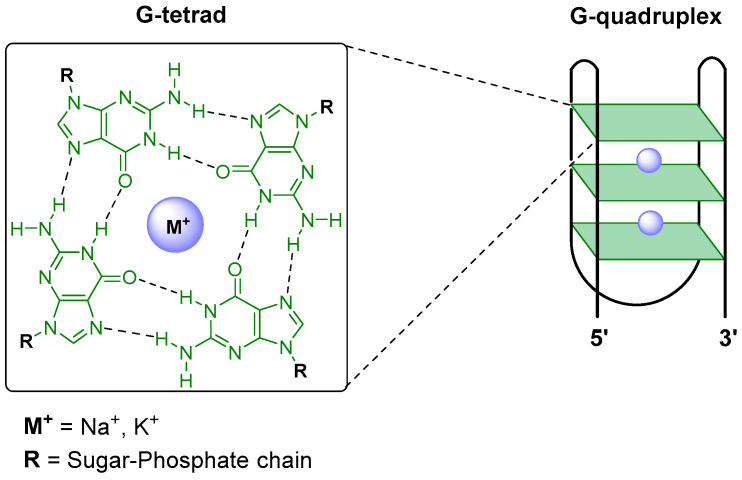
Schematic representation of a DNA G-quadruplex structure stabilized by a mono-cation.

G-quadruplexes (G4) are unusual four-stranded secondary structures of guanine-rich sequences of nucleic acids. The stacking of two or more guanine tetrads, which are formed by the Hoogsteen hydrogen bonding of four guanine bases held in a planar arrangement, gives rise to a thermodynamically stable knot-like G4 structure [138]. G4 are stabilized by the presence of monovalent cations such as potassium or sodium coordinated to the O6 atom of each G in the stacked G-tetrads (Figure 4).

These structures are observed throughout the genomes of several eukaryotic species [138,139,140,141] and, in mammals, are present in the promoter regions of regulatory genes and transcription factor binding sites, as well as in 5′ untranslated regions (5′ UTR) and telomeres [142,143]. The high prevalence of putative quadruplex-forming sequences (PQSs) in the genome of protozoan parasites, especially in kinetoplastid parasites [144,145,146,147], suggests that G4s are a key transcriptional regulator of these parasites [142]. In *Leishmania*, whose genome is particularly GC-rich (59.6% in *L. major*) among trypanosomatids (46.8% in *T. brucei*), 16,988 observed quadruplexes (OQs) were found versus 3231 for *T. brucei* [138]. The studies showed that *T. brucei* OQs are enriched in 5′UTR regions or gene promoters. In contrast, *Leishmania* showed depletion at these and other (e.g., exons, 3′UTR) intragenic regions, and no enrichment at non-coding regions.

Recent studies have established that *Leishmania* spp. display an unconventional DNA replication program, in which the timing of the DNA replication completion is chromosome length-dependent and relies on stochastic initiation events localized in regions with high AT content and increased G4 levels [148,149]. These findings reinforce the idea that targeting G4 structures in these pathogens could have deleterious consequences for the parasite.

**Figure 5:**
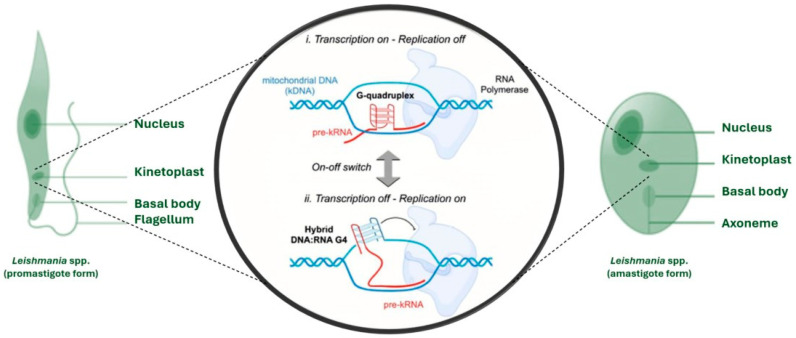
Proposed model for the “on–off” modulation of kinetoplast DNA (kDNA) transcription and replication mediated by G-quadruplex structures in *Leishmania* spp. The scheme illustrates a hypothetical regulatory mechanism in which G-quadruplex formation may act as a molecular switch between transcription and replication processes. It is conceptually adapted from Monti and Di Antonio [142] (©Wiley-VCH GmbH under license CC BY 4.0) who have proposed this mechanism in *T. brucei*.

This high prevalence of G4s and the crucial roles they play in the regulation of vital processes of these organisms, including immune evasion and virulence [150], underscore G4 as a promising drug target in kinetoplastid parasites [142]. In particular, the formation of DNA/RNA hybrid G4 structures has been proposed to modulate kDNA transcription and replication in African trypanosomes [151]. Hence, G4 specific ligands, designed to selectively target hybrid DNA/RNA quadruplexes, could potentially suppress transcription, whereas enhancing the formation of RNA G-quadruplexes might instead promote transcription, as depicted in Figure 5 [142].

In general, G4 ligands are molecules with large (planar shape) hydrophobic aromatic cores that can bind efficiently to G-tetrads by π–π stacking. These molecules usually have cationic groups to stabilize the electrostatic interaction with the G4 phosphate backbone regions and some side chains or substituents that can interact with the G4 loops and improve the selectivity towards G4 structures (reducing double-stranded DNA intercalation) [152,153]. With hundreds of G4 ligands described in the literature, it has become clear that, although planarity is essential for G4 binding, the design of molecules with higher flexibility and cationic side chains of refined lengths can improve the affinity and selectivity towards specific G4 topological arrangements [152].

**Figure 6:**
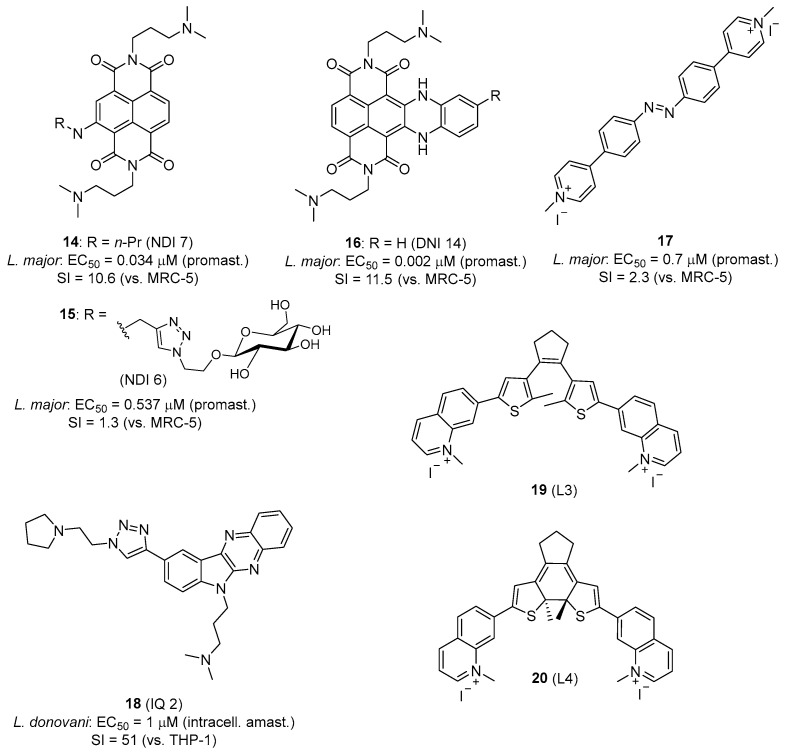
DNA G-quadruplex binders active against *T. brucei, T. cruzi*, and *Leishmania*.

Recent studies reported the potential of G4 ligands to treat HAT and leishmaniasis by targeting PQSs in the *T. brucei* and *Leishmania* genomes [147,154,155,156,157]. Belmonte-Reche et al. conducted a search for PQSs in the genomes of *T. brucei*, *L. major*, and *Plasmodium falciparum*. A highly abundant repeated sequence called EBR1 (5′-GGGCAGGGGGTGATGGGGAGGAGCCAGGG-3′) was identified in *T. brucei,* the G4-forming capacity of which was confirmed by biophysical methods [147]. The study also evaluated carbohydrate-naphthalene diimide ligands (carb-NDI), which were shown to bind EBR1 and displayed antiparasitic activity with IC_50_ in the nanomolar range against *T. brucei* and *L. major* (e.g., **14** and **15**, Figure 6) [147]. Confocal microscopy studies confirmed the localization of **14** and **15**, mainly in the nucleus and in the kinetoplast.

In subsequent studies, more potent NDI derivatives were developed and tested against *T. brucei* and *Leishmania*. Benassi et al. showed that core-extended NDI G4 ligands such as **16** had potent low nanomolar activity against *T. brucei* (IC_50_ = 0.26 nM; SI = 88 versus MRC-5 cells), while activity against *L. major* promastigotes was more modest (IC_50_ = 0.002 μM; SI = 11.5 versus MRC-5 cells) (Figure 6). For this series, a correlation emerged between *T. brucei* antiparasitic activity and the affinity for *T. brucei* G4 [158].

Azobenzene derivatives are another class of G4 ligands with excellent activity against *T. brucei* (IC_50_ = 0.7 nM, SI = 2286 versus MRC-5 cells) and submicromolar IC_50_ value against *L. major* promastigotes (**17**, Figure 6). In this case, the low selectivity index towards *Leishmania* (SI = 2.3) is due to the relatively high cytotoxicity (CC_50_ = 1.6 µM) of the compound on MRC-5 cells [159]. This study highlighted the importance of the relative position of the positively charged N atoms in the structure of these compounds for G4 stabilization and selectivity.

Karmakar et al. reported a host-directed therapeutic strategy using G4 ligands to modulate the host immune response against *Leishmania*; for example, the indolo [2,3-*b*]quinoxaline derivative IQ2 (**18**, Figure 6) stabilizes the c-MYC G4 promoter in macrophages, which causes the suppression of c-MYC transcription and translation. As a result, this compound exerted immunomodulatory effects (“host-directed therapy”) shifting the macrophage polarization from the disease-promoting M2 phenotype to the host-protective M1 phenotype. Compound **18** showed potent antileishmanial activity against promastigotes (IC_50_ = 0.18 µM) and intramacrophage amastigotes of *L. donovani* (IC_50_ = 1 µM) with low toxicity to THP-1 host cells [160].

G-quadruplexes have also been proposed as a therapeutic target for Chagas disease. Approximately 174 PQSs per 100,000 nucleotides were identified in the genome of *T. cruzi* [144]. Among a series of fourteen G4 ligands based on the dithienyl ethene (DTE) skeleton synthesized by Pérez-Soto et al., compounds **19** and **20** (Figure 6) achieved low micromolar activity against trypomastigotes of *T. cruzi* (IC_50_ = 1.5 and 3.3 μM, respectively), with good selectivity indexes (SI = 25 and 40, respectively). Biophysical studies showed that these compounds bind to different quadruplex sequences found in the genome of *T. cruzi* such as RCr (5′-GGGGACGGGAATGGGGGTGCATGAGGGG-3′), MCr (5′-GTGGAGGGGGAGGGTCATGGGG-3′), and TCr (5′-GGGAGGGACGGATGGGCAGAAACGGG-3′). In addition, fluorescence microscopy revealed the localization of the compounds in the nucleus and kinetoplast of the parasites after 24 h incubation, indicating that G4s are a potential target of these molecules [144].

**Figure 7:**
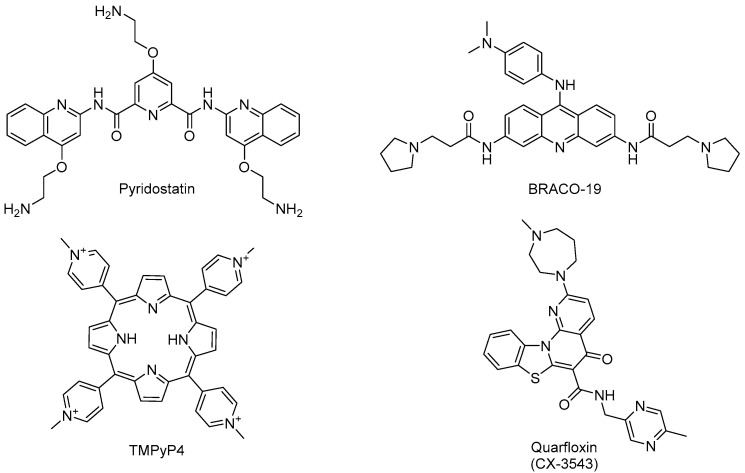
Examples of “classical” G-quadruplex binders active against protozoan parasites.

In the studies mentioned above, other “classical” G4 ligands initially used in cancer research such as pyridostatin, BRACO-19, and TMPyP4 were also tested against these parasites (Figure 7). However, these typical ligands showed much higher IC_50_ values against *T. brucei*, *T. cruzi* [144] or *Leishmania* [147], underscoring that it is possible to design selective antiparasitic G4 ligands (e.g., **14**, **15**) to target kinetoplastid parasites [147]. This trend was confirmed in another study on *P. falciparum.* Calvo and Wasserman established that TMPyP4 inhibits telomerase activity (IC_50_ = 5 µM) in this apicomplexan parasite, although it had a moderate effect on parasite growth (FCB-2 strain) at this concentration [161].

In the apicomplexan parasite *P. falciparum*, DNA G-quadruplexes can be detected in the nucleus of the parasite, which has an extremely AT-rich DNA (>80%) and therefore possesses few guanine-rich sequences with the potential to form G4s [162]. Notwithstanding, the 3D7 strain of *P. falciparum* is sensitive to several G-quadruplex-stabilizing drugs, including the RNA polymerase I (Pol I) inhibitor quarfloxin (Figure 7), an antitumoral G4 drug that also displays trypanocidal activity (IC_50_ = 155 nM) against *T. brucei* [141,163].

**Figure 8:**
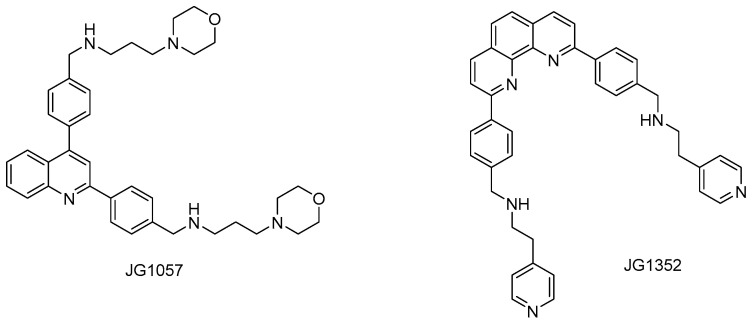
Examples of G-quadruplex binders active against helminths.

Recently, G4 formation was experimentally confirmed for sequences found in different parasitic helminths [139,140]. Small molecules able to selectively recognize G4 were found to bind to *Schistosoma mansoni* G4 motifs, and two of these ligands (JG1057 and JG1352, Figure 8) demonstrated potent activity against both larval and adult stages of this parasite [139].

In the last years, several studies reported the capacity of well-known MGBs to bind G4 structures. For instance, the diamidine drug diminazene (Figure 2), used for the treatment of animal trypanosomiasis, can bind G4 structures in a highly selective manner [164,165,166]. A recent study by Scott and Chalikian showed that MGBs such as netropsin and Hoechst 33258 can stabilize G-quadruplex-duplex hybrid (QDH) structures containing a hairpin duplex as a step-loop into the G4 core [167]. These studies suggest that (di)cationic minor groove binding molecules should be reassessed as G4 ligands, and may be used as templates for the design of new compounds with the sequence-specific recognition of G4s and/or QDH structures.

### 4.3. Mitochondrion-Targeted Small Molecules

Trypanosomatids are single-cell parasites with only one large mitochondrion containing essential enzymes involved in the energy-producing machinery. Hence, the mitochondrion is a critical organelle involved in the survival of these parasites. Accumulating evidence from our group [53,168,169,170,171,172,173,174] and others [175,176,177] demonstrates that conjugating antiparasitic agents with lipophilic cations is a highly effective chemotherapeutic strategy for selective mitochondrial targeting in protozoan parasites. However, because mitochondria-targeting strategies rely on conserved bioenergetic features shared with host cells, a careful evaluation of host mitochondrial toxicity remains essential for translational development. Among these, the triphenylphosphonium (TPP^+^) cation—characterized by a positive charge delocalized over a large and hydrophobic surface area—enables efficient electrophoretic transport across lipid bilayers driven by transmembrane potentials without the need for transporters. This targeting strategy has been reviewed by Zielonka et al. [178]. Another article by Cotman and co-workers nicely illustrates the recent advances in the development of mitochondrion-targeted molecules (MTMs), mainly TPP^+^-based and nitrogen-based heterocyclic MTMs, for different applications [179]. In particular, they highlight the effect of structural and physicochemical properties of MTMs on delivery and intrinsic bioactivity of the same.

**Figure 9:**
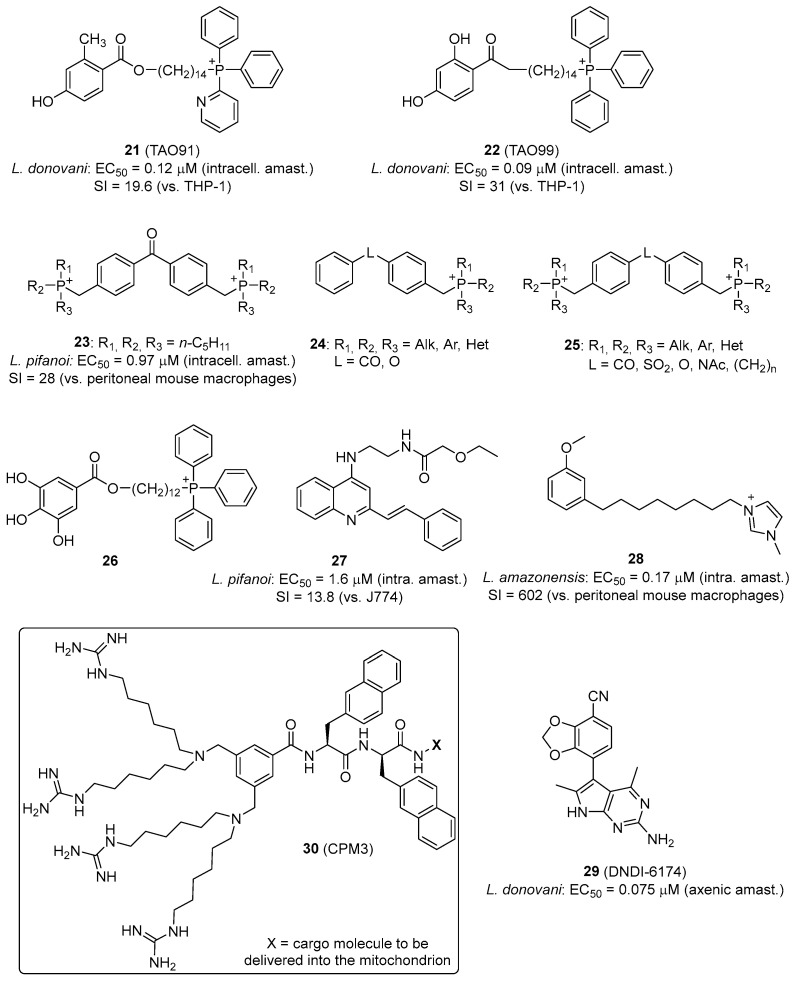
Examples of mitochondrion-targeted small molecules active against *Leishmania*. Frame: example of nonpeptidic cell-penetrating motif (CPM3).

In *Leishmania*, energy metabolism is mostly based on oxidative phosphorylation which accounts for approximately 75% of ATP production in *L. donovani* promastigotes, the rest being furnished by glycolysis [180]. In contrast, the bloodstream-form trypomastigotes of *T. brucei* obtain their energy via glucose-dependent respiration using a unique cytochrome-independent terminal oxidase, the trypanosome alternative oxidase (TAO) [181]. In recent years, our research group has established an innovative approach to enhance the antitrypanosomal efficacy of TAO inhibitors based on the 2,4-dihydroxybenzoic acid scaffold [172]. Given that TAO is localized on the matrix-facing surface of the mitochondrial inner membrane of African trypanosomes, we exploited mitochondrion-targeting approaches to improve drug delivery [172,181]. Using TPP^+^, quinolinium and pyridinium cations, we were able to boost the antiparasitic activity of several structurally simple TAO inhibitors [169,170,171,182]. For instance, the methylene-linked 2-methyl-4-hydroxybenzoate 2-pyridinyldiphenylphosphonium derivative **21** (Figure 9) is the first example of an allosteric inhibitor of TAO with broad-spectrum nanomolar range activity against African trypanosomes [168].

Interestingly, this class of MTMs also displays leishmanicidal activity in vitro (**21**) and in vivo (**22**). Compound **22** is a good example of a mitochondrion-targeted phosphonium salt with in vivo efficacy (>95% reduction in parasite load in spleen and liver) in a mouse model of visceral leishmaniasis by oral administration. Mechanistic investigations demonstrated that compound **22** permeates the plasma membrane and selectively localizes within the mitochondrion of *Leishmania* parasites. This mitochondrial targeting induced profound alterations in bioenergetic homeostasis, evidenced by a marked reduction in intracellular ATP concentrations, mitochondrial membrane potential collapse, and the substantial generation of reactive oxygen species [183].

Other bisphosphonium compounds have been shown to display antitrypanosomal and antileishmanial activity, interfering with the mitochondrion of the parasites to produce their effect (Figure 9) [53,174]. Benzophenone-derived bisphosphonium salts **23** exhibit leishmanicidal activity through the inhibition of succinate dehydrogenase (respiratory complex II) [53]. In *T. brucei*, mono- (**24**) and bisphosphonium (**25**) compounds inhibit the hydrolytic activity of the mitochondrial F_o_F_1_ ATPase [173].

Cortes et al. reported a series of TPP^+^–gallate conjugates (**26**) active against *T. cruzi*, the activity of which was mediated by the uncoupling effect of the gallic acid pharmacophoric group and not by the inhibition of a specific enzyme of the electron transport chain [176]. The uncoupling effect of TPP^+^ carriers on oxidative phosphorylation has been studied by Kulkarni et al. [184]. They found that Hückel charge has a stronger impact than lipophilicity on the uncoupling activity of TPP^+^ conjugates. Despite higher lipophilicity, 4-CF_3_-TPP^+^ derivatives showed no membrane depolarization, highlighting the inertness of this moiety and its suitability as a mitochondrion-targeting scaffold [184].

The quinoline scaffold has also been used as a mitochondrion-targeting moiety, providing compounds with antileishmanial activity. For instance, 4-aminostyrylquinolines such as compound **27** (Figure 9) have an increased basicity at the heterocyclic nitrogen and a positive charge delocalized by resonance across the pyridine ring. Hence, these molecules behave as delocalized lipophilic cations, which allows them to cross the lipid bilayers easily without the need for transporters. Compound **27**, which was active against intracellular amastigotes of *Leishmania pifanoi* in the low micromolar range, localized in the mitochondrion of the parasite [185]. Other quinolinium derivatives [186] and alternative cationic groups, such as imidazolium (**28**) and ammonium salts, were shown to display potent antiparasitic effect involving a mitochondrial target [175].

Another validated target of *Leishmania* is the cytochrome bc_1_ complex of the parasite’s electron transport chain. DNDI-6174 (**29**, Figure 9) is a promising pyrrolopyrimidine-derived preclinical candidate for visceral leishmaniasis that targets the cytochrome bc1. This compound is a promising drug candidate not only for *Leishmania*, but also for Chagas disease if combined with benznidazole [187].

Other mitochondrion delivery approaches exist apart from the “classical” mitochondrion-targeting scaffolds mentioned above. Appiah Kubi et al. reported a nonpeptidic cell-penetrating motif (**30**, Figure 9) consisting of four guanidinium groups and one or two hydrophobic naphthalene groups linked through a central scaffold for the specific delivery of both membrane-permeable small molecules and membrane-impermeable peptidyl cargoes into the mitochondrial matrix of mammalian cells [188,189]. Although this platform has not been used for antiparasitic compound delivery, it would deserve further study.

## 5. Nanomedicine-Based Strategies in Antileishmanial Therapy

Recent advances position nanomedicine as a compelling complement to conventional chemotherapy for leishmaniasis, with the explicit goals of enhancing targeted drug delivery, improving pharmacokinetic and biodistribution profiles, and circumventing mechanisms of drug resistance [190]. Liposomal formulations—exemplified clinically by liposomal amphotericin B—provide a proof of concept for efficient intracellular delivery, increasing macrophage uptake while reducing systemic toxicity [191]. Polymeric and metallic nanoparticles are another example of formulations that have demonstrated the effective targeted delivery of antimonials, amphotericin B, and miltefosine, diminishing host toxicity and mitigating efflux-mediated resistance pathways. The characteristics, advantages and limitations of such nanoparticles (NPs) have been reviewed recently [192,193].

Nanoemulsions and other nanocarriers are under active investigation for their capacity to cross biological barriers and to deliver therapeutics into parasitophorous vacuoles within infected macrophages, thereby improving intramacrophage bioavailability and antiparasitic activity [194,195]. However, despite promising preclinical and early clinical data, the translation of nanomedicine to widespread clinical use is constrained by challenges in scalable manufacturing, physicochemical and biological stability, regulatory requirements, and cost-effectiveness [193].

Nanomedicine can improve drug pharmacokinetics and the overall risk–benefit ratio by using functionalized nanocarriers for intracellular targeting, thereby increasing subcellular specificity while reducing systemic toxicity and treatment costs [196]. By tuning physicochemical properties and surface ligands, nanoparticles are selectively internalized and trafficked to specific organelles, enabling targeted accumulation and greater therapeutic precision [197]. Evidence from oncology and neurodegeneration indicates that mitochondrial targeting enhances therapeutic efficacy by promoting the selective accumulation of lipophilic cationic carriers driven by the organelle’s negative inner membrane potential, while amphiphilic mitochondriotropic motifs and targeting sequences facilitate endosomal escape and precise organelle delivery [198,199]. These studies have shown that, to deliver a bioactive molecule into the mitochondrial matrix, the nanoparticle-based delivery system must have a precise size, a lipophilic surface, a positive charge and a specific density of targeting ligands on its surface that allows for the recognition of and transport to the mitochondria [200]. Representative organelle-directed platforms—MITO porters and PLGA-*b*PEG-TPP^+^ systems, gold nanostars decorated with pro apoptotic peptides, and polymeric conjugates such as folate coated or TPP^+^ modified chitosan nanoparticles—illustrate how rational nanoformulations can provoke mitochondrial dysfunction and cancer cell death [201,202]. Mitochondrial-targeting principles can be applied to kinetoplastid parasites because the kinetoplast is a distinct mitochondrial subdomain essential for parasite survival [203]; mitochondrion-directed nanocarriers functionalized with targeting ligands or peptides therefore constitute a plausible strategy to deliver leishmanicidal agents with unprecedented subcellular specificity and to overcome the limited organelle selectivity of current drugs. Although most studies on mitochondrial targeting have been conducted in the context of neurodegenerative diseases and cancer, it has been demonstrated that nanoparticle accumulation within mitochondria enhances therapeutic efficacy while minimizing systemic toxicity [200,204,205,206,207]. These advances provide a conceptual framework that could be translated into the treatment of parasitic diseases such as leishmaniasis.

The active targeting of NPs offers significant potential for enhancing the specificity and efficacy of therapeutic delivery; however, its success is profoundly influenced by interactions with the biological milieu. Following systemic administration, NPs rapidly adsorb biomolecules to form a protein corona, which effectively redefines their surface characteristics. This corona can obscure engineered targeting ligands, altering cellular recognition and biodistribution, and often preventing the particles from reaching the intended cells or organs. Although positively charged NPs may facilitate mitochondrial uptake at the cellular level in vitro, in vivo they are highly susceptible to opsonization, aggregation, and nonspecific accumulation, particularly in organs such as the lungs. To mitigate these challenges, surface modifications such as PEGylation are widely employed to mask surface charges, reduce protein adsorption, and prolong circulation time. Therefore, rational nanoparticle design must integrate strategies to control corona formation, as it is a critical determinant of targeting efficiency, cellular uptake, and overall therapeutic performance. Nanoparticles that recruit specific serum proteins, such as apolipoproteins within the protein corona, have been shown to display enhanced targeting and biodistribution in vivo, with apolipoprotein-enriched coronas improving delivery efficiency in murine tumor models [208] and broader studies indicating that the modulation of corona composition can be strategically used to prolong circulation and optimize therapeutic outcomes [209].

Considering that *Leishmania* resides within the endolysosomal compartment of macrophages, it may be more effective to functionalize nanoparticles with macrophage-specific ligands to selectively deliver compounds targeting the kinetoplast. The effective delivery of nanocarriers to intracellular parasites such as *Leishmania* requires not only macrophage targeting but also trafficking toward acidic phagolysosomal compartments, where the parasitophorous vacuole is formed. This can be achieved by deliberately exploiting biological uptake pathways, including controlled opsonization to enhance FcγR/CR3-mediated phagocytosis [210], mannose receptor-mediated internalization using mannosylated surfaces [211], macrophage membrane cloaking to improve homing and uptake [212], and pH-responsive materials that trigger drug release within acidic endolysosomal environments [213,214]. Together, these strategies illustrate how rational nanodesign can exploit host cell biology to achieve predictable intracellular targeting and enhanced antiparasitic efficacy. This approach may provide a more precise and therapeutically relevant strategy than functionalizing nanoparticles solely for mitochondrial targeting, highlighting the importance of tailoring nanoparticle design to the subcellular localization and biology of the target pathogen.

### 5.1. Functionalized Mitochondrion-Targeted Nanoparticles

Unlike human cells, which contain multiple dynamic and functional mitochondria with free mtDNA dispersed in the cytoplasm, the protozoa of the order Kinetoplastida possess a single branched mitochondrion, indispensable for the survival of these trypanosomatids, making this subcellular structure a particular therapeutic target. This mitochondrion is distinctively characterized by the presence of the kinetoplast, which, as previously mentioned, is a specialized structure that houses the mtDNA in a highly condensed network of maxicircles and minicircles.

Despite its potential, the kinetoplast poses significant challenges as a therapeutic target, mainly due to its intracellular localization within the parasite’s single mitochondrion. Functionalized nanoparticles represent a new generation of intelligent delivery systems engineered for intracellular targeting in mitochondrial therapy [205,206,207]. These nanostructures can be selectively recognized, internalized, and accumulated within specific subcellular compartments, depending on their design features. From this perspective, the development of mitochondrion-targeted nanoparticles offers a promising strategy for delivering agents with selective activity against the kinetoplast of *Leishmania* spp.

**Figure 10:**
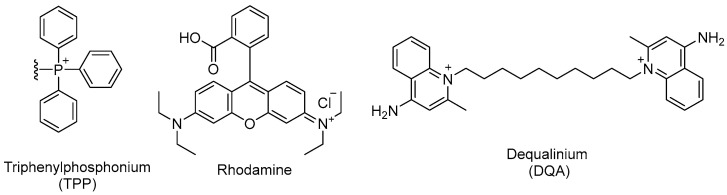
Examples of cationic ligands used for the functionalization of nanoparticles for specific delivery to mitochondria.

Many mitochondrial-targeting strategies exploit the highly negative potential of the inner mitochondrial membrane (IMM). This electrochemical gradient enables the accumulation of cations such as triphenylphosphonium, guanidinium, dequalinium, and rhodamine derivatives within the mitochondrial matrix (Figure 10). Most of these ligands are delocalized lipophilic cations with a positive charge, promoting electrostatic interactions with the anionic phospholipids of mitochondrial membranes and subsequent internalization (see Section 4.3) [215,216]. When conjugated with nanoparticles, these ligands allow for more precise drug delivery into mitochondria. 

In eukaryotic cells, the MMP is a dynamic parameter that can change depending on the metabolic state, nutrient availability, and energy demands of the cell, and it is distributed among multiple functional mitochondria [217]. In kinetoplastids, the MMP is maintained even during life cycle stages when oxidative phosphorylation is no longer the primary source of ATP [218]. Since the MMP is an essential component of mitochondrial function and viability in eukaryotic cells (including protozoa), the selectivity of a mitochondrial-targeted nanosystem would not be based exclusively on qualitative differences in membrane potential, but rather on the parasite’s structural and functional dependence on a single organelle per cell.

Thus, nanosystems can promote high drug concentrations within intracellular mononuclear phagocyte system infected cells. Once internalized, certain nanocarriers undergo intracellular disassembly, enabling the targeted release of their cargo into mitochondrial compartments, including the kinetoplast of *Leishmania* spp. Collectively, these features position nanometric drug delivery systems as powerful platforms for improving antileishmanial efficacy and inform the rational design of next-generation therapeutics targeting parasite-specific mitochondrial structures [219]. Below, we summarize the principal classes of mitochondrial-targeted nanocarriers, originally developed in oncology and other mitochondrion-centered therapies, such as neurodegenerative diseases, and discuss their relevance to antiparasitic drug development.

#### 5.1.1. Polymeric Nanoparticles

Polymeric nanoparticles are colloidal particles self-assembled from amphiphilic polymers that are highly biocompatible, minimally toxic, and capable of encapsulating therapeutic agents within their polymeric matrix or conjugating them on their surface. Their size typically falls within the submicron range (100–1000 nm). However, nanoparticles most effective in mitochondrial therapies are those <100 nm with a positive zeta potential, a condition that favors mitochondrial uptake [200]. This delivery system represents a multifunctional tool for the design of targeted therapies due to its ability to encapsulate bioactive agents, enable controlled release, and allow versatile surface functionalization to reach specific cellular compartments. Common polymeric designs in *Leishmania* include lipophilic blocks of polylactic acid (PLA), polyglycolic acid (PGA), poly(lactic-co-glycolic acid) (PLGA), and polycaprolactone (PCL), in combination with hydrophilic polyethylene glycol (PEG) blocks [220,221,222]. The latter provides greater stability, prolongs systemic circulation, and allows the conjugation of mitochondrion-targeting ligands (Table 2) [223]. Among these, the lipophilic cation triphenylphosphonium (TPP^+^, Figure 10), whose three benzene rings provide structural versatility for the incorporation of functional moieties [174], has been widely employed (see Section 4.3). In the context of *Leishmania* spp., accumulation within the cell can reach up to 10-fold-higher levels due to the parasite’s plasma membrane potential. Hence, this functionalization should enable nanoparticles to cross the parasite cell membrane, escape endolysosomal sequestration after internalization, and finally penetrate the highly negative double mitochondrial membrane of *Leishmania* spp. [207,224,225]. Such a strategy may offer significant advantages over conventional drugs by improving parasite specificity and reducing host toxicity.

#### 5.1.2. Liposomes and DQAsomes

Liposomes are vesicular structures composed primarily of phospholipids and cholesterol [223]. They present a great functional adaptability and can be structurally modified to reach subcellular compartments such as mitochondria. Their aqueous core and one or more lipid bilayers allow them to encapsulate hydrophilic, lipophilic, and macromolecular drugs. Liposomes are widely employed in drug delivery owing to their biocompatibility (i.e., biodegradable and low toxicity), their dual-loading capacity that facilitates combination therapies, and their ability to improve the bioavailability of drugs with distinct pharmacokinetic properties [205,223]. In the context of leishmaniasis, the most successful example is the liposomal formulation of amphotericin B (AmBisome^®^, Foster City, CA, USA) used as first-line therapy for VL. Studies on liposomal amphotericin B formulations have shown that toxicity and efficacy against cutaneous and visceral leishmaniasis can be finely tuned by controlling lipid composition, membrane rigidity, and drug aggregation state. Rigid, cholesterol-containing liposomes with slow drug release exhibit low toxicity and high efficacy, whereas more fluid liposomes favor faster release and improved bioavailability for the topical and oral treatment of CL [237]. Among other examples of liposomal formulations of antileishmanial drugs, we can cite the following: the incorporation of meglumine antimoniate into anionic liposomes, thereby improving its antileishmanial efficacy while minimizing its cytotoxicity to macrophages [238,239]; PEGylated liposomes loaded with meglumine antimoniate, which present greater stability and time in circulation allowing prolonged distribution of the drug [240]; or topical liposomes containing miltefosine for the treatment of *Leishmania major* infection [241]. However, challenges regarding intracellular internalization efficiency, in vivo stability, and mitochondrial specificity persist, making their optimization an ongoing area of research [242].

The surface functionalization of liposomes is key for mitochondrial targeting. Such design must consider interactions with the mitochondrial membrane and intracellular trafficking mechanisms. Liposomes functionalized with TPP^+^ are able to preserve vesicular integrity while gaining the capacity to cross the cell membrane and localize to the mitochondrial matrix due to their strong affinity for the mitochondrial inner membrane [243,244]. Nonetheless, the systemic application of permanently positively charged mitochondrial-targeting moieties such as TPP^+^ may be constrained by unfavorable pharmacokinetic behavior. The strong lipophilic cationic character of these ligands promotes extensive binding to plasma proteins and nonspecific interactions with cellular membranes, which in turn enhances opsonization and uptake by the mononuclear phagocyte system (MPS), leading to rapid clearance from circulation and reduced half-life in vivo [245,246,247]. To overcome this, negatively charged polymers such as PEG are often used to shield the surface, thereby enhancing circulation time and improving the ability of these carriers to cross biological barriers [248]. However, PEGylated liposomes can be recognized and cleared by anti-PEG antibodies upon repeated administration [249], a phenomenon known as accelerated blood clearance [250]. As a result, PEG is increasingly being replaced by biocompatible (i.e., biodegradable, non-toxic, and non-immunogenic) natural polymers such as hyaluronic acid, providing effective protection for cationic ligands [251].

Rhodamine derivatives (Figure 10) are a class of mitochondriotropic dyes with lipophilic and cationic properties that confer high affinity for mitochondrial membranes, enabling their specific localization and accumulation within the organelle. Similar to other lipophilic cations, this accumulation is membrane-potential dependent, a feature that has been extensively exploited in bioimaging to identify metabolically active cells, to measure mitochondrial membrane potential, and as a delivery strategy in oncology [252,253]. For example, Biswas et al. showed that the surface modification of liposomes with rhodamine-123-conjugated polymers resulted in enhanced cellular uptake and facilitated penetration across the inner mitochondrial membrane, resulting in higher cytotoxicity towards cancer cells (Table 2) [229]. Moreover, rhodamine offers advantages as a biological tracer due to its commercial availability, low cost, high quantum yield, and minimal interference with underlying metabolic processes [254]. In the context of *Leishmania* spp., this versatility enables the experimental validation of the mitochondrial tropism of nanoparticles, while simultaneously functioning as an active ligand to transport antileishmanial agents directly into the mitochondrial microenvironment.

Dequalinium (DQA) is a dicationic amphiphilic compound composed of two quinaldinium rings linked by a decamethylene chain (Figure 10). This molecular architecture allows DQA to self-assemble into vesicular structures resembling liposomes, known as DQAsomes [228,255]. DQAsomes were the first mitochondrion-targeted vesicular nanocarriers shown to selectively accumulate in the mitochondrial matrix [255]. Their enhanced accumulation and retention are attributed to the amphiphilic nature of DQA, which enables the formation of liposome-like structures capable of transporting drugs and DNA via nonspecific endocytic pathways, driven by the highly negative electrochemical gradient of the mitochondrial membrane [200,256]. This unique combination of structural and electrochemical properties makes DQAsomes a versatile platform for localized drug encapsulation and release (Table 2).

#### 5.1.3. Metallic Nanoparticles

Metallic nanoparticles hold great potential for the treatment and diagnosis of mitochondrial disorders due to their surface charges and capacity to conjugate with drugs, antibodies, and proteins, which not only protects them against host immune clearance but also increases their circulation time. They are typically composed of noble metals such as gold (Au) and silver (Ag), or metal oxides such as ZnO and Fe_3_O_4_. Their nanoscale dimensions confer a high surface-to-volume ratio, resulting in unique physicochemical properties, such as a large surface area per unit volume that exposes more functional groups on the surface, increasing their reactivity, adsorption, and interaction with biological molecules [257,258,259]. For instance, silver nanoparticles (AgNPs) have demonstrated antimicrobial activity, while gold nanoparticles (AuNPs) have been used to facilitate the translocation of drugs across cellular membranes.

These nanoparticles exhibit high structural stability and versatility for surface functionalization. Particle sizes between 5 and 260 nm are generally suitable for mitochondrial uptake [216]. However, their toxicity and potential to generate reactive oxygen species (ROS) in vital organs represent significant drawbacks in terms of selectivity [260]. Interestingly, this same ROS-generating capacity has been exploited in cancer therapy to selectively damage mitochondria of malignant cells and induce apoptosis. These effects are closely related to nanoparticle size and morphology, with fibrous or elongated nanoparticles larger than 400 nm being particularly difficult to eliminate from the body [260].

In the context of *Leishmania* spp., the physicochemical features of metallic nanoparticles, particularly their ability to generate ROS, could be strategically harnessed to induce selective damage to kDNA, provided that collateral host cell toxicity is minimized through specific mitochondrial targeting. This approach could improve both efficacy and safety profiles.

Iron oxide nanoparticles (IONPs), in particular, have demonstrated safe and effective action on mitochondria in cancer cells, overcoming challenges such as drug resistance and adverse side effects of conventional therapies. Their superparamagnetic properties enable their external guidance toward specific sites using magnetic fields, while their capacity to catalyze Haber–Weiss reactions allows the production of large amounts of ROS in cancer cells [206,216]. In the context of *Leishmania* spp., the physicochemical features of metallic nanoparticles, particularly their ability to generate ROS, could be strategically harnessed to induce selective damage to kDNA, provided that collateral host cell toxicity is minimized through specific mitochondrial targeting. This approach could improve both efficacy and safety profiles. The antileishmanial photodynamic therapeutic potential of ferromagnetic iron oxide nanorods was studied by Islam et al. [261]. They showed that these NPs induced oxidative stress in promastigotes and amastigotes of *L. tropica*, compromising parasite viability via apoptosis [261]. According to Rivas-García et al. [206], nanoparticles between 2 and 4 nm in size are optimal for entering subcellular compartments such as mitochondria.

### 5.2. Targeting Platforms Based on Mitochondrial Receptors and Transporters

The vast majority of mitochondrial proteins are encoded by the nuclear genome and require specialized translocation systems for their import into the organelle. Although the mitochondrial protein import systems of trypanosomatids and mammals share certain similarities, only a limited number of subunits of their machineries are conserved. These differences highlight the potential of this system, which is crucial for parasite survival and infection, as a promising drug target in trypanosomatids [262]. Chemical approaches to attack this import system could benefit from the mitochondrion-targeting strategies mentioned here.

In addition to strategies based on exogenous ligands or universal peptides, broadly acting cell-penetrating or organelle-targeting sequences have been widely employed to enhance intracellular drug delivery through conserved physicochemical mechanisms rather than cell-specific recognition. Classical cell-penetrating peptides (CPPs), such as TAT or poly-arginine, promote uptake across diverse cell types via endocytosis and membrane translocation [263]. Likewise, mitochondrial-targeting peptides (MTPs) exploit the universally conserved mitochondrial membrane potential and the TOM/TIM import machinery to drive matrix accumulation [264], while delocalized aromatic–cationic sequences such as the Szeto–Schiller peptides preferentially partition into mitochondrial membranes through electrostatic and lipophilic interactions [265]. Although these approaches efficiently improve intracellular and mitochondrial localization, their lack of inherent parasite specificity may lead to off-target effects in host cells. Consequently, there is growing evidence that endogenous components unique to trypanosomatids such as *Leishmania* could be exploited as selective markers or entry points for nanoparticles. These receptors and transporters, located in the kinetoplast and associated with essential survival processes of the parasite, not only could enhance mitochondrial specificity but also open new opportunities for the design of innovative therapeutic nanoplatforms. The most relevant components are summarized in Table 3, along with their function and potential applications in nanomedicine.

In summary, kinetoplastid mitochondrial transporters and receptors provide endogenous targets for directing nanoparticles to the mitochondria of *Leishmania*. Understanding their function, substrate specificity, and mitochondrial-targeting signals enables the rational design of more precise and effective drug delivery strategies, while minimizing off-target effects in host cells.

## 6. Conclusions and Future Perspectives

The kinetoplast represents a uniquely vulnerable and highly selective therapeutic target in *Leishmania* spp., integrating essential mitochondrial functions related to DNA maintenance, bioenergetics, redox balance, and parasite survival. Its distinctive molecular organization, together with the presence of a single mitochondrion in kinetoplastid parasites, provides a strong biological rationale for subcellularly targeted antileishmanial strategies capable of overcoming the major limitations of current chemotherapy, including systemic toxicity, limited selectivity, and the emergence of drug resistance [3,6].

Recent advances in molecular parasitology, medicinal chemistry, and nanotechnology have converged to enable innovative therapeutic approaches that exploit kinetoplast-specific vulnerabilities. As highlighted throughout this review, successful clinical translation will depend on integrating molecular pathophysiology with delivery strategies adapted to intracellular parasites and compatible with endemic, low-resource settings.

### 6.1. Integration of Molecular Pathophysiology and Innovative Therapies

Mitochondrial dysfunction is a central driver of cellular pathophysiology, as extensively demonstrated in cancer models, where alterations in bioenergetics, redox homeostasis, mitochondrial membrane potential, and genome integrity critically determine cell survival and therapeutic vulnerability [256]. Similar principles apply to *Leishmania* parasites, whose single mitochondrion integrates energy production, kinetoplast DNA (kDNA) maintenance, and oxidative stress control, rendering it particularly susceptible to targeted perturbation. Pathophysiological features such as an elevated mitochondrial membrane potential, limited antioxidant capacity, and reliance on kinetoplast-associated processes create exploitable vulnerabilities that can be selectively targeted without affecting host cells.

The therapeutic strategies discussed in this review—such as DNA minor groove binders, G-quadruplex ligands, and mitochondrion-targeted small molecules—which are summarized in Table 4, illustrate how detailed knowledge of parasite molecular biology can be translated into rational drug design [21,142]. Accumulating experimental evidence demonstrates that interference with kinetoplast-associated processes—including kDNA replication, topoisomerase activity, G-quadruplex formation, mitochondrial membrane potential maintenance, and redox homeostasis—leads to profound mitochondrial dysfunction and irreversible parasite death [38,53,62]. These tightly interconnected processes rely on molecular components that are absent or highly divergent in mammalian cells, providing a solid basis for selective pharmacological intervention. Collectively, these data position the kinetoplast not only as a validated drug target but also as a conceptual platform for mechanism-driven antiparasitic drug discovery.

In particular, mitochondrion-targeted compounds exploiting the strong negative membrane potential of the parasite enable preferential intramitochondrial accumulation, resulting in enhanced antiparasitic efficacy while limiting host toxicity [53,174]. When combined with nanomedicine-based delivery systems, these approaches further improve intracellular trafficking, subcellular specificity, and pharmacokinetic profiles, providing a realistic framework for repurposing existing chemotypes and accelerating the development of next-generation antileishmanial agents [190,196].

However, designing nanoparticles capable of selectively targeting the mitochondria of the parasite without affecting the host cell mitochondria is inherently challenging. Mitochondria are highly conserved organelles across eukaryotic cells, sharing similar membrane structures, surface proteins, and electrochemical gradients. This structural and functional similarity makes it difficult for nanoparticles to discriminate between host and parasite mitochondria once internalized. Furthermore, because *Leishmania* resides within the endolysosomal compartment of macrophages, nanoparticles must reach this compartment to access the parasite. Strategies that rely on general mitochondrial-targeting motifs (e.g., positive charges or lipophilic cations) may enhance uptake by all mitochondria within the host cell, increasing the risk of cytotoxicity. Consequently, indirect targeting approaches—such as directing nanoparticles to the host cell compartment containing the parasite—are often more feasible and safer than attempting to discriminate mitochondria directly [270].

### 6.2. Multidisciplinary Relevance and Dermatological Implications

Leishmaniasis is a paradigmatic neglected tropical disease that requires multidisciplinary solutions integrating parasitology, medicinal chemistry, pharmaceutical technology, immunology, and clinical dermatology. This need is particularly evident in cutaneous and mucocutaneous leishmaniasis, where parasite persistence within skin macrophages leads to chronic inflammation, tissue destruction, permanent scarring, and significant psychosocial burden [12].

Cutaneous involvement is not limited to visible lesions but is associated with prolonged disease duration, residual scarring, pain, pruritus, secondary infections, and a profound impairment of quality of life, as consistently captured by dermatology-specific outcome measures such as the Dermatology Life Quality Index. These dermatological manifestations underscore the need for therapeutic strategies capable of achieving efficient intralesional parasite clearance while minimizing tissue damage and long-term cosmetic and psychosocial sequelae [271]. Kinetoplast-directed therapies are especially relevant in the dermatological context, as they enable the selective elimination of intracellular amastigotes within skin lesions while minimizing systemic exposure. Nanoparticle-based delivery systems offer promising opportunities for topical, intralesional, or macrophage-targeted administration, potentially improving lesion resolution, reducing treatment duration, and enhancing patient adherence [192,195].

For the treatment of visceral leishmaniasis via parenteral administration, the localization of the parasite in the liver, spleen, and bone marrow generally aligns with the passive accumulation patterns of conventional nanoparticles, due to the fenestrated endothelium in these organs that allows nanoparticles to extravasate and reach resident macrophages [272]. However, in cutaneous leishmaniasis, infected macrophages reside in the dermis, a compartment poorly accessible to nanoparticles following either parenteral or topical administration. This limitation explains why formulations such as AmBisome are more effective for visceral leishmaniasis than cutaneous leishmaniasis [273,274], and why topical application has not been successful. Even in lesioned skin, it is difficult for nanoparticles, including very small ones, to reach the dermal layer intact and effectively interact with infected macrophages [275,276]. Therefore, for nanoparticles to be effective in the local treatment of cutaneous leishmaniasis, they must either be administered intralesionally or be designed to enhance drug diffusion and penetration, rather than relying on mitochondrial targeting. Hence, in the specific context of cutaneous leishmaniasis, drugs with intrinsic specificity for the parasite (i.e., kinetoplast) may be more effective than nanoparticles designed solely for mitochondrial targeting, emphasizing that rational therapeutic design should prioritize biological specificity over complex nanoparticle engineering.

Beyond dermatology, the principles discussed in this review are broadly applicable to other kinetoplastid diseases, reinforcing the value of shared molecular targets and delivery platforms across *Leishmania*, *T. cruzi*, and *T. brucei* infections [3,21]. From a global health perspective, translating kinetoplast-focused strategies into affordable and scalable therapies will require the early integration of formulation science, manufacturing feasibility, and regulatory considerations, together with sustained collaboration between academic, clinical, and public health stakeholders.

In conclusion, kinetoplast-directed therapies exemplify a mechanism-driven and subcellularly precise approach to antiparasitic drug development. By integrating molecular insight with innovative chemical and nanotechnological strategies, this framework offers a promising path toward safer, more effective, and context-appropriate treatments for leishmaniasis and other neglected tropical diseases. Moreover, such platforms may facilitate combination strategies integrating direct antiparasitic activity with host-directed immunomodulation by enabling the co-delivery of kinetoplast-targeted agents and immunomodulatory cues within infected macrophages, thereby simultaneously promoting parasite clearance and the restoration of effective local immune responses [16,160].

## Figures and Tables

**Table 2:** Examples of mitochondrion-targeted NPs and their targeting mechanisms.

Vehicle	Ligand	Cargo	References
Polymeric nanoparticle	PLGA-b-PEG- TPP^+^	ionidamide, α-tocopheryl succinate, crucumin	[226]
Polymeric nanoparticle	PLGA-b-PEG- TPP^+^	coenzyme Q10	[227]
Polymeric nanoparticle	Dequalinio	curcumin	[228]
Liposomes	Rh123	paclitaxel	[229]
Liposomes	stearyl-TPP (STPP^+^)	sclareol	[230]
Liposomes	STPP^+^	ceramide	[231]
Liposomes	Dequalinio and TPGS1000(D-alfa-tocoferol polietilglicol 1000)	topotecan	[232]
DQAsomes	Dequalinio	curcumin	[233]
DQAsomes	Dequalinio	gapmer antisense oligonucleotide	[234]
DQAsomes	Dequalinio	doxorubicin	[235]
Solid lipid nanoparticle	TPP^+^	*Ficus religiosa* L. extract	[236]

**Table 3:** Examples of mitochondrial receptors that could be targeted with functionalized NPs.

Receptor	Function	Potential Application for NP Functionalization
RICB8A [RNA Import Complex (RIC)]	Subunit of the RIC: - receptor for type II tRNAs - participates in tRNA import machinery - linked to complex III of the respiratory chain [266]	NPs could be functionalized with type II tRNA fragments or aptamers to target this receptor and promote mitochondrial import.
RIC1/F1α	Subunit of the RIC: - receptor for type I tRNAs - essential for cytosolic RNA import and for the generation of ATP-dependent MMP - shows high affinity for the TψC arm and anticodon regions of tRNAs, which are critical for import [266,267]	NPs may be functionalized with type I tRNA fragments or aptamers to interact with RIC1 at the inner mitochondrial membrane, favoring active import. Alternatively, the tRNA-dependent ATPase activity of RIC1 could be exploited to design responsive systems that trigger drug release upon type I tRNA recognition.
LMIT1 [*Leishmania* Mitochondrial Iron Transporter 1]	*L. amazonensis* mitochondrial iron importer - homologous to mitoferrin transporters - essential for survival and virulence - supports differentiation into amastigotes - protects parasite against oxidative stress [268]	Functionalization of NPs with siderophore-like moieties or iron carriers may enable recognition by LMIT1, promoting NP uptake or localized drug release. Moreover, the upregulation of LMIT1 during specific life stages could be leveraged to improve selectivity.
LmABCB3 [Atypical mitochondrial ABC transporter]	*L. major* mitochondrial transporter - involved in heme biosynthesis and iron–sulfur cluster biogenesis essential for parasite viability - contains an N-terminal extension required for mitochondrial targeting [269]	NPs could be functionalized with ligands mimicking its natural substrates (heme or derivatives) and/or peptides based on its mitochondrial targeting sequence (specific N-terminal amino acid motifs), enabling selective drug delivery into the parasite mitochondria and enhancing specificity and efficacy.

**Table 4:** From target to translation: kinetoplast-directed therapeutic strategies for leishmaniasis.

Target/Strategy	Representative Compounds or Platforms	Parasite Stage Affected *^a^*	Reported Activity (IC_50_)	Selectivity/Host Cytotoxicity	In Vivo Evidence	Major Limitations
kDNA Minor groove binders	Diamidines	P; IA	μM–nM depending on scaffold; less active against IA	Variable	Limited in vivo studies for investigational compounds	Membrane permeability barriers; host toxicity; transporter dependence
	BisAIA (DB766, JNII40)	P; IA	<1 μM *L. donovani* <5 μM *L. Major*	Often improved vs. diamidines SI > 1000 (macrophages)	Reported in rodent models (e.g., DB766)	In vivo toxicity depending on the scaffold; unsymmetrical bisAIA are active but toxic
	S-MGB (219)	IA	1 μM *L. donovani*	N/A	N/A	Inhibit hERG channel
Topoisomerase inhibitors (TopoI/TopoII)	Voacamine, diindolylmethane (DIM), indolylmaleimides, CT3, holanamine	P; IA	Low μM IC_50_ for several compounds	Some parasite selectivity vs human topoisomerases	Mouse models reported for some compounds	Limited clinical development; potential off-target effects
G-quadruplex stabilizers	Naphthalene diimides (NDI)	P	<1 μM *L. major*	SI < 15 (MRC-5 cells)	Limited in vivo validation	Cytotoxicity; insufficient selectivity; limited pharmacokinetic data; poor membrane permeability

*^a^* P = promastigotes, IA = intracellular amastigotes.

## Data Availability

No new data were created or analyzed in this study. Data sharing is not applicable to this article.
